# Epistaxis

**Published:** 1909-05-15

**Authors:** 


### Laryngology and Rhinology.

### EPISTAXIS.

Hæmorrhage from the nose may be due primarily to various systemic diseases, to certain alterations of environment, or to local affections. Constitutional diseases cause bleeding in two ways, either by raising the blood-pressure or by producing alterations in the quality or coagulability of the blood; but it is not possible in every case to determine by which method the effect has been produced.

### General Conditions.

Epistaxis is frequently associated with such diseases as chronic nephritis, arteriosclerosis, cirrhosis of the liver; with blood-diseases such as purpura, scurvy, the severe anæmias, lucocythæmia, and hæmophilia; and with acute fevers such as enteric fever, pneumonia, scarlet fever, and measles. It is distinctly uncommon in cases of venous engorgement due to cardiac and pulmonary disease, even when associated with a marked degree of cyanosis. It occurs also in certain conditions which cannot be described as diseases, for it is especially common in delicate growing children about the age of puberty without any obvious pathological cause, and it is a frequent accident among persons going rapidly into a rarefied atmosphere, as in balloon ascents and mountain climbs. Nose-bleeding is very common in persons of the so-called plethoric habit, and it is sometimes stated that it indicates a liability to apoplexy, but it is at least doubtful if this is actually the case.

The epistaxis of these diseases may be slight or very profuse, and may recur repeatedly from one or both or alternate nostrils until the patient shows serious signs of loss of blood; but it is seldom fatal, and rarely even dangerous, except in the severer forms of profound alterations of the blood. In hæmophilia the nose is the commonest of all sites for bleeding, and the slow, continuous dripping is highly characteristic. Of the acute fevers, enteric is peculiarly associated with epistaxis.

### Local Affections.

In healthy people the commonest cause of epistaxis is a condition of dry rhinitis. The nasal mucous membrane has the task of moistening all the inspired air, and has to provide a large amount of aqueous vapour for the purpose. If the secreting power of the glands falls a little below normal, the mucous lining becomes too dry, and this occurs especially where the current of air first impinges—namely, at an area on the septum within an inch of the nostril. If this part of the septum project slightly to one side, the effect is there increased, and, in point of fact, the bleeding spot is often found at the apex of such a projection. Several small vessels may often be seen on this region, and the hæmorrhage usually occurs from a minute erosion on one of these. When the mucosa becomes dry, a small crust of dried mucous and dust tends to adhere to the spot, and, on forcible removal by picking or violent blowing of the nose, leaves a tiny erosion which is deepened and extended by repetition of the process.

Two other local causes of bleeding are of some importance, for though uncommon they may cause profuse and repeated hæmorrhage, and should not be overlooked. The first is the so-called "bleeding polypus of the septum," which is a purplish rounded growth, varying in size from a pin's head to a bean, histologically an angeioma, and springing from the region of the septum, already mentioned as the common site for epistaxis. The second is largely explained by its title "hereditary multiple telangiectases," and has attracted much attention of late; it is characterised by the presence of minute telangiectases scattered over the cheeks, mouth, and mucous membrane of the nose, fauces, and palate, which bleed on the slightest provocation. Hæmorrhage from the nose also accompanies severe ulceration and necrosis and malignant growths; it should be noted that it is not a symptom of the ordinary mucous polypus, and that spontaneous bleeding from an apparently simple polypus should arouse the suspicion of malignancy. Bleeding, of course, is common after injury, especially accompanied by fracture, and severe recurrent hæmorrhage is fairly frequent after surgical procedures, especially when vaso-constrictors, such as adrenalin, have been freely used. In cases of epistaxis, also, due to constitutional disease the bleeding is nearly always from the anterior part of the septum, but it may occur from any part or be a general oozing, especially in hæmophilia and the blood-diseases. Brown Kelly refers to cases of hæmorrhage occurring from the region above the anterior end of the middle turbinal body.

### Treatment.

In the large majority of cases, epistaxis occurring from the front part of the septum, may be temporarily arrested by inserting a plug of gauze or wool for an inch within the nostril, and making pressure on it with the finger on the ala of the nose. After the hæmorrhage has been arrested the bleeding point should be sealed with the cautery at a dull red heat. The minute erosion is often indistinguishable, and it is often wise to re-start the bleeding by gentle stroking with a probe, and then to arrest it by a pledget of wool soaked in cocaine solution before using the cautery. It should be remembered that hæmorrhage is liable to recur from a neighbouring spot or from the opposite side. Bleeding from the deeper parts, as after operation or in severe constitutional diseases, demands plugging of the nose. Rose's tampon is troublesome and often unsatisfactory; and plugging the posterior nares, as with Bellocq's sound, should never be performed, for the blood collected in the nasal cavities penetrates into the accessory sinuses, and may readily infect them. The plugging must always be done by introducing a strip of ribbon gauze with fine nasal forceps, and should produce an even and regular pressure. Powerful styptics must on no account be employed, and the use of adrenalin causes subsequent vaso-dilatation, and is inadvisable. Peroxide of hydrogen has none of these disadvantages, and the gauze may well be dipped in a five-volume solution of this drug before insertion. It must never be left for more than 36 hours, but must then be removed, and, if necessary, replaced. Otherwise the dangers of sepsis are considerable, and include severe inflammations of the middle ear. The gauze should be removed very gently after thorough moistening, preferably with peroxide of hydrogen. In many cases of severe bleeding restlessness and a rapid pulse are prominent features, and an injection of morphia is of the greatest value. In hæmophilia, and wherever the coagulability of the blood is diminished, calcium lactate in doses of 15 to 30 grains every four hours is recommended, though its action is not yet definitely proved. Of minor measures, the patient should be kept quiet in bed with the head raised, iced water may be applied to the face, and hot bottles to the feet and trunk.

